# Supplementary material for: RNA sequencing reveals niche gene expression effects of beta-hydroxybutyrate in primary myotubes
Source: Life Sci Alliance. 2021 Aug 18;4(10):e202101037. doi: 10.26508/lsa.202101037 (PMC8380668; doi:10.26508/lsa.202101037)
Supplement: Supplementary file 1 [file LSA-2021-01037_TableS1.docx]

| **Gene name** | **log2FC** | **FDR.BH** |
| --- | --- | --- |
| *Gm43590* | -4,46 | 3,8E-03 |
| *Gm48113* | -4,21 | 2,2E-02 |
| *Clec4a1* | -4,09 | 1,9E-04 |
| *Gm49339* | -3,93 | 2,2E-02 |
| *Gm10645* | -3,68 | 2,2E-02 |
| *Bcl2a1d* | -3,52 | 7,6E-04 |
| *Tifab* | -3,47 | 2,9E-04 |
| *Cd300ld* | -3,45 | 5,0E-04 |
| *Apoc2* | -3,45 | 2,1E-02 |
| *AW112010* | -3,32 | 1,1E-02 |
| *Gm29100* | -3,24 | 7,5E-04 |
| *Ggt5* | -3,08 | 8,9E-03 |
| *Osm* | -3,06 | 1,7E-03 |
| *Pf4* | -3,05 | 1,5E-03 |
| *Gm21188* | -3,03 | 1,3E-03 |
| *B430306N03Rik* | -2,99 | 5,6E-03 |
| *Mfng* | -2,94 | 1,4E-03 |
| *Prdm1* | -2,88 | 3,0E-03 |
| *Lrrc25* | -2,87 | 8,4E-04 |
| *Tnfsf8* | -2,86 | 1,1E-02 |
| *Gpr34* | -2,86 | 2,0E-03 |
| *Cd86* | -2,83 | 8,8E-03 |
| *Acod1* | -2,82 | 2,5E-02 |
| *Tnfsf13* | -2,82 | 9,0E-03 |
| *H2-Aa* | -2,81 | 8,3E-03 |
| *3930402G23Rik* | -2,76 | 3,1E-02 |
| *Rab19* | -2,76 | 2,4E-02 |
| *Aoah* | -2,75 | 1,6E-03 |
| *Mcoln2* | -2,74 | 4,5E-03 |
| *Card9* | -2,74 | 6,1E-03 |
| *Apol7c* | -2,70 | 4,4E-03 |
| *H2-Eb1* | -2,67 | 4,6E-04 |
| *P2ry13* | -2,64 | 2,7E-03 |
| *Siglece* | -2,62 | 3,4E-02 |
| *Slfn5* | -2,61 | 2,7E-02 |
| *Cd74* | -2,60 | 1,5E-02 |
| *Kcnj10* | -2,59 | 4,6E-04 |
| *Clec10a* | -2,57 | 2,6E-02 |
| *Abcg3* | -2,54 | 5,0E-04 |
| *Ccr2* | -2,54 | 8,8E-03 |
| *P2ry12* | -2,51 | 3,4E-03 |
| *Gmfg* | -2,51 | 1,7E-03 |
| *Ptpro* | -2,51 | 2,5E-02 |
| *Inpp5d* | -2,50 | 2,8E-02 |
| *Tpbgl* | -2,50 | 8,9E-04 |
| **Gene name** | **log2FC** | **FDR.BH** |
| *A630001G21Rik* | -2,48 | 4,1E-03 |
| *Gpr171* | -2,47 | 2,1E-02 |
| *Gm5431* | -2,46 | 8,0E-03 |
| *A130077B15Rik* | -2,43 | 6,8E-03 |
| *Psmb8* | -2,43 | 1,6E-02 |
| *Themis2* | -2,41 | 9,1E-03 |
| *Clec7a* | -2,40 | 2,1E-03 |
| *Gna15* | -2,37 | 4,4E-02 |
| *Naip6* | -2,36 | 7,3E-03 |
| *Fcgr2b* | -2,36 | 1,8E-03 |
| *Srgn* | -2,34 | 4,6E-04 |
| *Itgax* | -2,34 | 1,6E-03 |
| *Fcgr4* | -2,33 | 5,6E-03 |
| *Ms4a4a* | -2,33 | 5,7E-03 |
| *H2-Ab1* | -2,33 | 3,2E-02 |
| *Slamf8* | -2,33 | 3,7E-02 |
| *Cx3cr1* | -2,31 | 8,9E-04 |
| *Gm11992* | -2,31 | 7,3E-03 |
| *Lst1* | -2,30 | 1,4E-02 |
| *Sncaip* | -2,28 | 8,9E-03 |
| *2210406H18Rik* | -2,27 | 1,1E-02 |
| *Clec4n* | -2,27 | 3,8E-03 |
| *Csf2rb* | -2,25 | 1,4E-02 |
| *Tlr9* | -2,25 | 2,6E-02 |
| *Icam2* | -2,24 | 3,7E-02 |
| *Apobec1* | -2,24 | 2,2E-04 |
| *Batf* | -2,24 | 2,9E-02 |
| *Tnfrsf11a* | -2,24 | 1,7E-03 |
| *4933430I17Rik* | -2,23 | 2,3E-02 |
| *Ms4a14* | -2,23 | 2,0E-03 |
| *C5ar1* | -2,22 | 1,7E-03 |
| *Spi1* | -2,21 | 8,8E-03 |
| *C1qa* | -2,21 | 1,8E-03 |
| *Drd1* | -2,21 | 1,3E-02 |
| *Clec4a3* | -2,21 | 1,6E-02 |
| *Cnr2* | -2,20 | 1,3E-02 |
| *Fgd2* | -2,19 | 7,3E-03 |
| *Fcrls* | -2,19 | 9,9E-04 |
| *Ms4a6c* | -2,18 | 4,6E-04 |
| *B3gnt7* | -2,15 | 7,6E-03 |
| *Cd244a* | -2,15 | 4,0E-02 |
| *Btk* | -2,15 | 6,0E-04 |
| *Siglec1* | -2,14 | 3,5E-02 |
| *Plcb2* | -2,14 | 2,1E-02 |
| *Cysltr1* | -2,13 | 1,7E-03 |
| **Gene name** | **log2FC** | **FDR.BH** |
| *Selplg* | -2,13 | 1,6E-02 |
| *Lyl1* | -2,12 | 3,7E-02 |
| *Tlr13* | -2,11 | 1,0E-03 |
| *Arhgap9* | -2,10 | 7,5E-04 |
| *Grap2* | -2,10 | 2,2E-02 |
| *Pik3cg* | -2,10 | 6,8E-03 |
| *Cd200r2* | -2,10 | 2,2E-02 |
| *Slc15a3* | -2,09 | 1,6E-02 |
| *C1qc* | -2,08 | 5,7E-04 |
| *She* | -2,07 | 3,7E-02 |
| *Tyrobp* | -2,06 | 7,5E-04 |
| *Il7r* | -2,06 | 2,3E-02 |
| *Rps2-ps13* | -2,05 | 1,6E-02 |
| *Cybb* | -2,01 | 2,6E-03 |
| *Fermt3* | -2,01 | 5,7E-04 |
| *Dock2* | -2,01 | 1,4E-03 |
| *Ccr1* | -2,00 | 7,2E-04 |
| *Vav1* | -2,00 | 1,6E-03 |
| *Ms4a6d* | -1,99 | 9,8E-04 |
| *Nlrp1b* | -1,99 | 3,3E-03 |
| *Ptprc* | -1,97 | 4,6E-03 |
| *C1qb* | -1,97 | 6,3E-04 |
| *Fcrl1* | -1,95 | 4,3E-02 |
| *Stap1* | -1,95 | 4,0E-02 |
| *Tnf* | -1,95 | 1,0E-02 |
| *Foxj1* | -1,94 | 3,3E-02 |
| *Grk3* | -1,93 | 5,0E-03 |
| *Napsa* | -1,92 | 2,2E-02 |
| *Cd52* | -1,92 | 2,1E-03 |
| *Irf8* | -1,90 | 1,4E-03 |
| *Cd33* | -1,90 | 3,8E-03 |
| *Slc11a1* | -1,90 | 1,4E-02 |
| *Gm37168* | -1,90 | 2,1E-02 |
| *Il10ra* | -1,89 | 4,9E-02 |
| *Gpr65* | -1,89 | 2,5E-02 |
| *Bcl2a1b* | -1,89 | 1,9E-03 |
| *Lair1* | -1,88 | 2,5E-02 |
| *Ebi3* | -1,88 | 3,6E-02 |
| *Runx3* | -1,88 | 8,1E-03 |
| *Tal1* | -1,88 | 4,1E-02 |
| *Csf1r* | -1,88 | 1,3E-03 |
| *Hcls1* | -1,87 | 2,2E-03 |
| *Cd300c2* | -1,87 | 1,1E-02 |
| *Csf2rb2* | -1,86 | 1,0E-02 |
| *Lpxn* | -1,86 | 4,5E-03 |
| **Gene name** | **log2FC** | **FDR.BH** |
| *Igsf6* | -1,86 | 1,7E-03 |
| *Stra6l* | -1,86 | 4,8E-02 |
| *Tnfaip8l2* | -1,86 | 2,8E-03 |
| *Cd83* | -1,85 | 1,6E-03 |
| *Casp1* | -1,85 | 1,0E-02 |
| *I830077J02Rik* | -1,85 | 1,4E-02 |
| *Bin2* | -1,84 | 3,3E-02 |
| *Coro1a* | -1,84 | 8,8E-03 |
| *Naip2* | -1,84 | 1,8E-02 |
| *Was* | -1,84 | 1,2E-02 |
| *Stab1* | -1,83 | 6,8E-03 |
| *Hk3* | -1,83 | 1,3E-02 |
| *Ctss* | -1,83 | 5,7E-04 |
| *Ccdc88b* | -1,82 | 4,4E-02 |
| *Myo1f* | -1,82 | 8,7E-04 |
| *Fcgr1* | -1,82 | 2,4E-02 |
| *Lcp1* | -1,80 | 1,4E-03 |
| *Gpr31c* | -1,80 | 1,3E-02 |
| *Styxl1* | -1,78 | 3,6E-02 |
| *Ikzf1* | -1,78 | 1,1E-02 |
| *Sirpb1c* | -1,78 | 2,2E-02 |
| *Trem2* | -1,77 | 7,5E-04 |
| *P2ry6* | -1,76 | 2,6E-03 |
| *Marchf1* | -1,75 | 2,1E-02 |
| *Plxnc1* | -1,75 | 1,7E-03 |
| *H2-DMb1* | -1,75 | 2,6E-02 |
| *Entpd1* | -1,75 | 2,3E-03 |
| *Ms4a7* | -1,74 | 1,7E-03 |
| *Snx20* | -1,74 | 1,1E-02 |
| *Cd84* | -1,74 | 8,2E-04 |
| *B3gnt8* | -1,73 | 1,9E-02 |
| *Arl11* | -1,73 | 7,7E-03 |
| *Gngt2* | -1,73 | 5,6E-03 |
| *Gpr162* | -1,72 | 1,3E-02 |
| *Fcgr3* | -1,72 | 2,2E-04 |
| *Fcer1g* | -1,72 | 1,8E-03 |
| *Pik3ap1* | -1,72 | 1,4E-02 |
| *Il16* | -1,71 | 2,7E-03 |
| *Tbxas1* | -1,71 | 1,7E-02 |
| *Mpeg1* | -1,71 | 1,4E-03 |
| *Itgb2* | -1,70 | 2,6E-03 |
| *Wfdc17* | -1,70 | 1,5E-02 |
| *Ptpn7* | -1,70 | 1,7E-03 |
| *Nlrc4* | -1,69 | 3,1E-02 |
| *Clec5a* | -1,69 | 1,1E-02 |
| **Gene name** | **log2FC** | **FDR.BH** |
| *Cd68* | -1,69 | 2,5E-03 |
| *Lilr4b* | -1,68 | 3,7E-02 |
| *Nuf2* | -1,68 | 3,9E-03 |
| *Arhgap25* | -1,67 | 6,5E-03 |
| *Dpep2* | -1,67 | 1,1E-02 |
| *Tlr1* | -1,66 | 4,5E-03 |
| *Rasgef1b* | -1,66 | 1,2E-02 |
| *C3ar1* | -1,66 | 7,5E-04 |
| *Dok3* | -1,65 | 1,9E-03 |
| *Rinl* | -1,65 | 4,2E-03 |
| *Pld4* | -1,65 | 3,2E-03 |
| *Cxcl16* | -1,64 | 1,7E-03 |
| *Alox5ap* | -1,63 | 2,1E-02 |
| *Evi2b* | -1,63 | 4,2E-02 |
| *Ccl9* | -1,63 | 3,0E-02 |
| *Aif1* | -1,63 | 8,8E-03 |
| *Pirb* | -1,63 | 5,0E-03 |
| *Shtn1* | -1,63 | 9,4E-03 |
| *AI662270* | -1,62 | 1,8E-02 |
| *Arhgap30* | -1,62 | 6,6E-03 |
| *Ltc4s* | -1,62 | 4,4E-02 |
| *Arhgap45* | -1,62 | 4,6E-04 |
| *Cd200r4* | -1,61 | 7,9E-03 |
| *Hck* | -1,60 | 5,0E-04 |
| *Tgfbi* | -1,60 | 3,4E-03 |
| *Angptl7* | -1,59 | 1,1E-02 |
| *Dock10* | -1,59 | 2,0E-03 |
| *Cd48* | -1,59 | 3,6E-02 |
| *Ncf1* | -1,58 | 3,0E-04 |
| *Neurl3* | -1,57 | 8,7E-04 |
| *Laptm5* | -1,57 | 5,9E-04 |
| *Tlr7* | -1,57 | 8,9E-03 |
| *Sash3* | -1,57 | 2,8E-02 |
| *Cyth4* | -1,56 | 5,7E-04 |
| *Nrros* | -1,56 | 8,8E-03 |
| *Ccl3* | -1,56 | 2,6E-02 |
| *Gm14548* | -1,55 | 1,8E-02 |
| *Mybl2* | -1,55 | 2,5E-02 |
| *Rasal3* | -1,55 | 3,0E-02 |
| *Plek* | -1,55 | 4,6E-04 |
| *Hrh1* | -1,54 | 4,4E-02 |
| *Lat2* | -1,53 | 1,7E-02 |
| *Adgre1* | -1,53 | 2,7E-02 |
| *Hvcn1* | -1,53 | 5,2E-03 |
| *Il1rn* | -1,52 | 3,4E-02 |
| **Gene name** | **log2FC** | **FDR.BH** |
| *Lyz2* | -1,52 | 9,8E-04 |
| *Otulinl* | -1,52 | 4,0E-02 |
| *Rassf6* | -1,52 | 4,7E-02 |
| *BE692007* | -1,51 | 4,3E-02 |
| *Itgam* | -1,51 | 1,3E-02 |
| *Ncf2* | -1,50 | 1,3E-02 |
| *Fgr* | -1,50 | 4,1E-02 |
| *Il2rg* | -1,50 | 3,8E-03 |
| *Tmem273* | -1,50 | 8,8E-03 |
| *Esco2* | -1,48 | 3,0E-02 |
| *Tmem106a* | -1,47 | 3,8E-03 |
| *Lcp2* | -1,47 | 3,5E-02 |
| *Kif18b* | -1,44 | 2,4E-02 |
| *Ptpn6* | -1,42 | 1,7E-03 |
| *Clec4d* | -1,40 | 1,4E-02 |
| *Sla* | -1,39 | 8,7E-03 |
| *Ccl6* | -1,38 | 5,5E-03 |
| *Adam8* | -1,38 | 2,2E-04 |
| *Fgl2* | -1,37 | 2,1E-02 |
| *Lgals3bp* | -1,36 | 7,7E-03 |
| *Cenpa* | -1,36 | 1,4E-02 |
| *Cd180* | -1,36 | 2,9E-02 |
| *Hmgb2* | -1,34 | 1,1E-02 |
| *Mmp13* | -1,34 | 2,9E-02 |
| *Fut1* | -1,34 | 4,9E-02 |
| *Icosl* | -1,33 | 3,4E-02 |
| *Arhgap4* | -1,32 | 4,7E-02 |
| *Cd93* | -1,31 | 2,9E-04 |
| *Cxcl14* | -1,30 | 2,4E-02 |
| *Cfp* | -1,28 | 8,1E-03 |
| *Mki67* | -1,27 | 2,1E-02 |
| *Fbxo5* | -1,26 | 2,1E-02 |
| *Ndc80* | -1,26 | 2,5E-02 |
| *AB124611* | -1,20 | 4,2E-02 |
| *Elmo1* | -1,19 | 2,7E-02 |
| *Cd14* | -1,19 | 2,1E-02 |
| *Top2a* | -1,18 | 1,1E-02 |
| *Apobr* | -1,16 | 3,7E-03 |
| *Lpcat2* | -1,16 | 2,2E-02 |
| *Tnfsf13os* | -1,16 | 3,2E-02 |
| *Mtfr2* | -1,16 | 3,0E-02 |
| *Cep55* | -1,15 | 2,1E-02 |
| *Ctla2b* | -1,13 | 2,7E-02 |
| *Duxbl1* | -1,13 | 2,2E-02 |
| *Cd300a* | -1,11 | 2,5E-02 |
| **Gene name** | **log2FC** | **FDR.BH** |
| *Syk* | -1,10 | 4,3E-04 |
| *Adap1* | -1,10 | 1,5E-02 |
| *Slamf9* | -1,10 | 1,6E-02 |
| *Gda* | -1,09 | 2,0E-03 |
| *Irf5* | -1,09 | 1,5E-02 |
| *Rnasel* | -1,08 | 3,4E-03 |
| *Anpep* | -1,06 | 2,5E-02 |
| *Fli1* | -1,06 | 3,8E-03 |
| *Nusap1* | -1,06 | 5,0E-02 |
| *3010003L21Rik* | -1,05 | 4,3E-02 |
| *Lfng* | -1,02 | 2,2E-02 |
| *Kif22* | -1,01 | 4,9E-02 |
| *Sirpa* | -1,00 | 2,6E-06 |
| *Mrc1* | -1,00 | 3,2E-02 |
| *H2-DMa* | -0,99 | 1,6E-03 |
| *Pik3r5* | -0,99 | 2,9E-02 |
| *Gpnmb* | -0,98 | 1,1E-03 |
| *Cd28* | -0,94 | 1,4E-03 |
| *Nckap1l* | -0,93 | 5,6E-03 |
| *Cotl1* | -0,92 | 2,2E-04 |
| *Gng2* | -0,92 | 1,7E-02 |
| *Atp6v0d2* | -0,89 | 1,3E-02 |
| *1700007L15Rik* | -0,89 | 2,9E-02 |
| *Il6ra* | -0,85 | 2,4E-02 |
| *Fabp5* | -0,85 | 5,7E-04 |
| *Hps1* | -0,84 | 1,4E-03 |
| *Slc16a6* | -0,84 | 2,0E-02 |
| *Glipr1* | -0,84 | 5,8E-03 |
| *Abcb1b* | -0,83 | 2,9E-02 |
| *Jade2* | -0,83 | 8,1E-03 |
| *Evi2a* | -0,80 | 1,6E-02 |
| *Kcnk2* | -0,80 | 4,9E-03 |
| *Cd53* | -0,79 | 1,4E-03 |
| *Ugt1a7c* | -0,75 | 3,4E-02 |
| *Plcg2* | -0,74 | 6,6E-03 |
| *H2-D1* | -0,73 | 3,0E-02 |
| *Arhgap19* | -0,71 | 7,5E-03 |
| *Abca1* | -0,70 | 7,3E-03 |
| *9630028I04Rik* | -0,70 | 4,1E-02 |
| *Fnip2* | -0,68 | 4,3E-03 |
| *Fignl1* | -0,68 | 2,6E-02 |
| *Vrk1* | -0,67 | 4,4E-02 |
| *Dkk2* | -0,67 | 2,9E-04 |
| *Dcx* | -0,66 | 4,6E-04 |
| *Aqp1* | -0,66 | 3,6E-02 |
| **Gene name** | **log2FC** | **FDR.BH** |
| *Mylk* | -0,66 | 1,7E-02 |
| *Rgs10* | -0,65 | 1,2E-02 |
| *Dhrs3* | -0,65 | 1,4E-03 |
| *Pla2g7* | -0,64 | 1,3E-02 |
| *Clrn1* | -0,64 | 2,4E-02 |
| *Rab32* | -0,63 | 4,4E-02 |
| *Lpl* | -0,63 | 3,5E-03 |
| *Igfbp4* | -0,62 | 3,4E-02 |
| *Cyp1b1* | -0,62 | 2,6E-02 |
| *4933421O10Rik* | -0,61 | 2,7E-02 |
| *Arhgdib* | -0,61 | 6,2E-03 |
| *Gzme* | -0,59 | 1,5E-02 |
| *Tcp11l1* | -0,59 | 2,7E-02 |
| *Rab7b* | -0,58 | 3,0E-02 |
| *Ctsc* | -0,58 | 5,0E-04 |
| *Lipa* | -0,55 | 2,6E-03 |
| *Gpr137b-ps* | -0,55 | 4,1E-03 |
| *Cacna2d2* | -0,53 | 1,5E-02 |
| *Hsd17b11* | -0,53 | 2,4E-02 |
| *P2rx4* | -0,53 | 1,9E-02 |
| *Casp8* | -0,52 | 2,5E-02 |
| *Stmn1* | -0,52 | 1,4E-03 |
| *Gm17455* | -0,52 | 2,0E-02 |
| *Ptgs1* | -0,51 | 1,0E-02 |
| *Tagln2* | -0,50 | 2,8E-03 |
| *Havcr2* | -0,50 | 4,6E-02 |
| *Man2b1* | -0,49 | 1,2E-02 |
| *Ptprj* | -0,48 | 2,5E-02 |
| *Fam20a* | -0,48 | 2,0E-02 |
| *Tep1* | -0,47 | 5,6E-03 |
| *Galnt7* | -0,47 | 4,9E-02 |
| *Dab2* | -0,46 | 1,7E-03 |
| *Gm2a* | -0,46 | 1,4E-03 |
| *Dock8* | -0,46 | 1,6E-02 |
| *Emb* | -0,45 | 2,3E-02 |
| *Tor4a* | -0,45 | 2,4E-02 |
| *Hexb* | -0,45 | 1,9E-02 |
| *Il10rb* | -0,45 | 2,6E-03 |
| *Col10a1* | -0,45 | 4,7E-02 |
| *Hmga2* | -0,44 | 9,4E-03 |
| *Kcnn4* | -0,44 | 4,5E-02 |
| *B2m* | -0,44 | 2,2E-02 |
| *Atr* | -0,43 | 2,7E-02 |
| *Poglut3* | -0,43 | 2,4E-02 |
| *Srsf3* | -0,43 | 7,5E-04 |
| **Gene name** | **log2FC** | **FDR.BH** |
| *Coro1c* | -0,43 | 1,1E-03 |
| *Dynap* | -0,42 | 2,7E-02 |
| *Arl6ip1* | -0,41 | 1,6E-02 |
| *Rhobtb1* | -0,41 | 2,1E-02 |
| *Ftl1* | -0,41 | 4,4E-02 |
| *Kctd12* | -0,41 | 1,3E-02 |
| *Rpa2* | -0,41 | 1,3E-02 |
| *Arpc1b* | -0,41 | 2,4E-02 |
| *Cnn2* | -0,40 | 1,6E-02 |
| *Casp6* | -0,40 | 2,6E-02 |
| *Sri* | -0,40 | 2,6E-02 |
| *Dock11* | -0,40 | 2,4E-02 |
| *Anxa8* | -0,39 | 2,0E-02 |
| *Arl4c* | -0,39 | 4,5E-02 |
| *Pnmt* | -0,39 | 4,5E-02 |
| *Pgm2* | -0,39 | 1,8E-02 |
| *Mymx* | -0,39 | 2,2E-03 |
| *Tmsb4x* | -0,38 | 9,1E-03 |
| *Tpd52* | -0,38 | 3,7E-03 |
| *Specc1* | -0,38 | 2,7E-02 |
| *Wdr75* | -0,38 | 9,9E-03 |
| *Ctsh* | -0,37 | 5,0E-02 |
| *Arpc5* | -0,37 | 2,4E-03 |
| *Edil3* | -0,37 | 2,1E-03 |
| *Gpr63* | -0,37 | 3,9E-02 |
| *G6pdx* | -0,37 | 1,7E-02 |
| *Wipf1* | -0,36 | 2,4E-02 |
| *Unc93b1* | -0,36 | 3,7E-02 |
| *Gusb* | -0,36 | 8,1E-03 |
| *Mylip* | -0,36 | 3,7E-02 |
| *Hgsnat* | -0,35 | 3,7E-02 |
| *Mafb* | -0,35 | 8,0E-04 |
| *Prkcd* | -0,35 | 7,6E-03 |
| *Swap70* | -0,34 | 4,2E-02 |
| *Ocrl* | -0,34 | 2,6E-02 |
| *Ctsb* | -0,34 | 3,4E-02 |
| *Slc7a8* | -0,33 | 4,2E-03 |
| *Sema4c* | -0,33 | 9,0E-03 |
| *Sptlc2* | -0,33 | 1,3E-02 |
| *Cdk4* | -0,33 | 6,0E-04 |
| *Bri3bp* | -0,32 | 4,3E-02 |
| *Tpbg* | -0,32 | 2,2E-02 |
| *Actb* | -0,32 | 1,6E-02 |
| *Ptk2b* | -0,31 | 1,6E-02 |
| *Utp20* | -0,31 | 3,0E-02 |
| **Gene name** | **log2FC** | **FDR.BH** |
| *Pip4k2a* | -0,31 | 1,7E-02 |
| *Dtx4* | -0,30 | 2,6E-02 |
| *Nt5m* | -0,30 | 3,0E-02 |
| *Manf* | -0,30 | 2,7E-02 |
| *Anxa1* | -0,29 | 5,7E-03 |
| *Iqgap1* | -0,29 | 2,7E-02 |
| *Mgp* | -0,29 | 4,9E-02 |
| *Sgpl1* | -0,29 | 8,2E-03 |
| *Zfp36l2* | -0,29 | 4,1E-02 |
| *Rab8b* | -0,28 | 1,6E-02 |
| *Itga6* | -0,28 | 9,0E-03 |
| *Actg1* | -0,28 | 4,9E-02 |
| *Rap2a* | -0,28 | 2,4E-02 |
| *Cers2* | -0,28 | 4,2E-02 |
| *Cndp2* | -0,28 | 3,4E-02 |
| *Hmgcr* | -0,28 | 1,9E-02 |
| *Txn1* | -0,28 | 1,6E-02 |
| *Shc4* | -0,27 | 3,7E-02 |
| *Vwa5a* | -0,27 | 1,1E-02 |
| *Hexa* | -0,27 | 7,3E-03 |
| *Grn* | -0,27 | 1,3E-02 |
| *Abhd12* | -0,27 | 1,4E-02 |
| *Psap* | -0,27 | 7,3E-03 |
| *Gnpda1* | -0,26 | 3,3E-02 |
| *Lasp1* | -0,26 | 3,8E-02 |
| *Cfl1* | -0,26 | 2,9E-02 |
| *Elmsan1* | -0,26 | 8,5E-03 |
| *Rai14* | -0,25 | 1,7E-02 |
| *Tmod3* | -0,25 | 1,8E-02 |
| *Hnrnpa1* | -0,25 | 2,1E-03 |
| *Marcks* | -0,25 | 2,6E-03 |
| *Ralb* | -0,25 | 3,9E-02 |
| *Gadd45a* | -0,24 | 1,3E-02 |
| *Rap2b* | -0,24 | 1,6E-02 |
| *Snx2* | -0,24 | 3,8E-02 |
| *Serp1* | -0,23 | 3,7E-02 |
| *Lgmn* | -0,23 | 3,7E-02 |
| *Dnajc3* | -0,21 | 3,8E-02 |
| *Calm2* | -0,21 | 3,8E-02 |
| *Gnai3* | -0,21 | 4,0E-02 |
| *Odc1* | -0,21 | 1,1E-02 |
| *Actr2* | -0,19 | 3,0E-02 |
| *Man1c1* | -0,18 | 3,8E-02 |
| *Rasa3* | -0,18 | 3,6E-02 |
| *Eif4a1* | -0,18 | 2,4E-02 |
| **Gene name** | **log2FC** | **FDR.BH** |
| *Mta2* | -0,15 | 3,0E-02 |
| *Dnm2* | 0,12 | 4,3E-02 |
| *Larp1* | 0,12 | 4,9E-02 |
| *Cs* | 0,14 | 2,2E-02 |
| *Carhsp1* | 0,14 | 2,7E-02 |
| *Mdh2* | 0,15 | 3,0E-02 |
| *Fxr2* | 0,16 | 4,1E-02 |
| *Npepps* | 0,16 | 1,6E-02 |
| *Inppl1* | 0,17 | 2,7E-02 |
| *Atmin* | 0,18 | 4,3E-02 |
| *Emc1* | 0,18 | 5,0E-02 |
| *Adgre5* | 0,18 | 3,4E-02 |
| *Aco2* | 0,18 | 2,1E-02 |
| *Stip1* | 0,18 | 2,5E-02 |
| *Ssbp3* | 0,19 | 2,2E-02 |
| *Prcc* | 0,20 | 2,4E-02 |
| *Ube2j2* | 0,20 | 3,9E-02 |
| *Aldoa* | -0,73 | 6,5E-01 |
| *Zswim8* | 0,21 | 3,5E-02 |
| *Irf2bp1* | 0,21 | 2,5E-02 |
| *Ppm1b* | 0,21 | 6,1E-03 |
| *Ankrd40* | 0,21 | 4,8E-02 |
| *Fam53a* | 0,21 | 3,2E-02 |
| *Kcmf1* | 0,21 | 2,4E-02 |
| *Opa3* | 0,21 | 1,4E-02 |
| *Rnf126* | 0,21 | 2,4E-02 |
| *Ints9* | 0,22 | 1,6E-02 |
| *Lrrc20* | 0,22 | 1,6E-02 |
| *Mtss2* | 0,22 | 3,5E-02 |
| *Ssx2ip* | 0,22 | 9,0E-03 |
| *L3mbtl3* | 0,22 | 3,7E-02 |
| *Stom* | 0,22 | 1,7E-02 |
| *Dcaf6* | 0,23 | 2,8E-02 |
| *Pik3c2b* | 0,23 | 3,2E-02 |
| *Coq9* | 0,23 | 2,6E-02 |
| *Map1b* | 0,23 | 3,9E-02 |
| *Tbx15* | 0,23 | 2,1E-02 |
| *Usp31* | 0,24 | 2,9E-02 |
| *Ttpal* | 0,24 | 4,6E-02 |
| *Cc2d1a* | 0,24 | 1,6E-02 |
| *Lrrn1* | 0,24 | 8,8E-03 |
| *Agap3* | 0,24 | 1,6E-03 |
| *Gab1* | 0,24 | 2,8E-02 |
| *Zfp322a* | 0,25 | 5,0E-02 |
| *Ubald1* | 0,25 | 2,9E-02 |
| **Gene name** | **log2FC** | **FDR.BH** |
| *Atg9a* | 0,25 | 2,1E-02 |
| *Arhgap44* | 0,25 | 2,4E-02 |
| *Hrc* | 0,25 | 3,6E-02 |
| *Ciao2b* | 0,25 | 2,4E-02 |
| *Hs6st1* | 0,26 | 4,9E-03 |
| *Fut11* | 0,26 | 4,2E-02 |
| *Cyc1* | 0,26 | 3,7E-02 |
| *Sema3d* | 0,26 | 3,9E-02 |
| *Glce* | 0,26 | 4,9E-02 |
| *Rai2* | 0,27 | 1,1E-02 |
| *Stbd1* | 0,27 | 1,4E-02 |
| *Mmp15* | 0,27 | 8,8E-03 |
| *Zfp365* | 0,28 | 1,4E-02 |
| *Atn1* | 0,28 | 2,5E-02 |
| *Slc38a2* | 0,28 | 7,5E-04 |
| *Iba57* | 0,28 | 4,9E-02 |
| *Fgfbp1* | 0,28 | 3,1E-02 |
| *Foxo6* | 0,29 | 2,0E-02 |
| *Trim3* | 0,29 | 8,9E-03 |
| *Gpatch8* | 0,29 | 3,9E-02 |
| *Pnmal2* | 0,29 | 2,2E-03 |
| *Adamtsl4* | 0,29 | 3,9E-02 |
| *Psg28* | 0,29 | 3,3E-03 |
| *Mtres1* | 0,30 | 2,1E-03 |
| *Fhl1* | 0,30 | 3,4E-02 |
| *Sall2* | 0,31 | 4,9E-02 |
| *Nfic* | 0,31 | 1,1E-02 |
| *Sema6c* | 0,31 | 4,2E-02 |
| *Hspa2* | 0,31 | 1,7E-02 |
| *6430548M08Rik* | 0,32 | 2,2E-03 |
| *Mafa* | 0,34 | 8,3E-03 |
| *Traf3ip3* | 0,34 | 3,2E-02 |
| *Fbxl16* | 0,34 | 8,1E-03 |
| *Hivep1* | 0,35 | 2,2E-02 |
| *Cstf2* | 0,36 | 2,1E-03 |
| *Fgf11* | 0,36 | 2,0E-03 |
| *Epb41l3* | 0,37 | 2,6E-02 |
| *E2f2* | 0,38 | 4,5E-03 |
| *Satb1* | 0,38 | 1,5E-02 |
| *Otub2* | 0,38 | 6,1E-03 |
| *Efna2* | 0,39 | 4,7E-02 |
| *Ckmt2* | 0,41 | 2,0E-02 |
| *Ache* | 0,44 | 2,6E-03 |
| *Per1* | 0,47 | 3,0E-02 |
| *Nfatc2* | 0,51 | 1,6E-03 |
| **Gene name** | **log2FC** | **FDR.BH** |
| *Zscan2* | 0,60 | 1,1E-02 |
| *1700013G24Rik* | 0,60 | 3,1E-02 |
| *Ankrd34a* | 0,60 | 3,2E-02 |
| *Kcna7* | 0,64 | 1,7E-03 |
| *Gm49540* | 0,70 | 3,1E-02 |
| *Rgs4* | 0,71 | 3,4E-02 |
| *Stc1* | 0,80 | 3,1E-02 |
| *Gngt1* | 0,84 | 3,5E-02 |
| *Gm50064* | 0,87 | 4,1E-02 |
| *Myh13* | 1,01 | 3,8E-02 |
| *Xlr3b* | 1,44 | 1,5E-02 |
| *Fam131c* | 1,47 | 1,6E-02 |
| *Gm16675* | 1,77 | 2,6E-02 |
| *Gm20554* | 1,84 | 3,5E-02 |
| *A230056P14Rik* | 1,89 | 4,2E-02 |
| *Gria4* | 2,37 | 3,8E-03 |
| *Zdhhc23* | 2,40 | 1,1E-02 |
| *Sowaha* | 2,46 | 1,9E-02 |
| *Rnf43* | 2,57 | 1,7E-02 |
| *Atp7b* | 2,79 | 3,7E-02 |
