# Supplementary material for: RNA sequencing reveals niche gene expression effects of beta-hydroxybutyrate in primary myotubes
Source: Life Sci Alliance. 2021 Aug 18;4(10):e202101037. doi: 10.26508/lsa.202101037 (PMC8380668; doi:10.26508/lsa.202101037)
Supplement: Supplementary file 2 [file LSA-2021-01037_TableS2.docx]

**Supplemental table 2: GSEA positively enriched pathways by βOHB treatment in primary myocytes.**

**NAME NES FDR q-val**

MMU00020.CITRATE.CYCLE..TCA.CYCLE..KEGG 2.35 0.000

MMU04710.CIRCADIAN.RHYTHM.KEGG 2.08 0.002

MMU01200.CARBON.METABOLISM.KEGG 1.92 0.012

MMU00190.OXIDATIVE.PHOSPHORYLATION.KEGG 1.91 0.010

MMU04260.CARDIAC.MUSCLE.CONTRACTION.KEGG 1.91 0.009

MMU01210.2.OXOCARBOXYLIC.ACID.METABOLISM.KEGG 1.88 0.010

MMU04714.THERMOGENESIS.KEGG 1.84 0.015

MMU01230.BIOSYNTHESIS.OF.AMINO.ACIDS.KEGG 1.82 0.015

MMU00270.CYSTEINE.AND.METHIONINE.METABOLISM.KEGG 1.81 0.016

MMU03015.MRNA.SURVEILLANCE.PATHWAY.KEGG 1.77 0.020

MMU00051.FRUCTOSE.AND.MANNOSE.METABOLISM.KEGG 1.74 0.023

MMU04152.AMPK.SIGNALING.PATHWAY.KEGG 1.73 0.022

MMU05012.PARKINSON.DISEASE.KEGG 1.71 0.025

MMU04922.GLUCAGON.SIGNALING.PATHWAY.KEGG 1.68 0.031

MMU00630.GLYOXYLATE.AND.DICARBOXYLATE.METABOLISM.KEGG 1.67 0.032

MMU04932.NON.ALCOHOLIC.FATTY.LIVER.DISEASE..NAFLD..KEGG 1.63 0.041

MMU05016.HUNTINGTON.DISEASE.KEGG 1.57 0.060

MMU00640.PROPANOATE.METABOLISM.KEGG 1.56 0.066

MMU04136.AUTOPHAGY...OTHER.KEGG 1.54 0.068

MMU00010.GLYCOLYSIS...GLUCONEOGENESIS.KEGG 1.50 0.092

MMU05010.ALZHEIMER.DISEASE.KEGG 1.50 0.089

MMU00620.PYRUVATE.METABOLISM.KEGG 1.48 0.090

MMU04120.UBIQUITIN.MEDIATED.PROTEOLYSIS.KEGG 1.48 0.089

MMU00534.GLYCOSAMINOGLYCAN.SYNTHESIS.HEPARAN.SULFATE.KEGG 1.47 0.092

MMU04910.INSULIN.SIGNALING.PATHWAY.KEGG 1.45 0.096
