# Supplementary material for: RNA sequencing reveals niche gene expression effects of beta-hydroxybutyrate in primary myotubes
Source: Life Sci Alliance. 2021 Aug 18;4(10):e202101037. doi: 10.26508/lsa.202101037 (PMC8380668; doi:10.26508/lsa.202101037)
Supplement: Supplementary file 3 [file LSA-2021-01037_TableS3.docx]

**Supplemental table 3: GSEA negatively enriched pathways by βOHB treatment in primary myocytes.**

**NAME NES FDR q-val**

MMU05150.STAPHYLOCOCCUS.AUREUS.INFECTION.KEGG -2.70 0.000

MMU04061.VIRAL.PROTEIN.INTERACTION.WITH. CYTOKINE.RECEPTOR.KEGG -2.50 0.000

MMU04145.PHAGOSOME.KEGG -2.49 0.000

MMU04142.LYSOSOME.KEGG -2.35 0.000

MMU04640.HEMATOPOIETIC.CELL.LINEAGE.KEGG -2.35 0.000

MMU05140.LEISHMANIASIS.KEGG -2.34 0.000

MMU05323.RHEUMATOID.ARTHRITIS.KEGG -2.29 0.000

MMU04380.OSTEOCLAST.DIFFERENTIATION.KEGG -2.27 0.000

MMU05332.GRAFT.VERSUS.HOST.DISEASE.KEGG -2.26 0.000

MMU04062.CHEMOKINE.SIGNALING.PATHWAY.KEGG -2.26 0.000

MMU05152.TUBERCULOSIS.KEGG -2.24 0.000

MMU04940.TYPE.I.DIABETES.MELLITUS.KEGG -2.23 0.000

MMU04514.CELL.ADHESION.MOLECULES..CAMS..KEGG -2.22 0.000

MMU04060.CYTOKINE.CYTOKINE.RECEPTOR.INTERACTION.KEGG -2.21 0.000

MMU00531.GLYCOSAMINOGLYCAN.DEGRADATION.KEGG -2.19 0.000

MMU05416.VIRAL.MYOCARDITIS.KEGG -2.17 0.000

MMU04672.INTESTINAL.IMMUNE.NETWORK.FOR.IGA.PRODUCTION.KEGG -2.16 0.000

MMU05330.ALLOGRAFT.REJECTION.KEGG -2.16 0.000

MMU04666.FC.GAMMA.R.MEDIATED.PHAGOCYTOSIS.KEGG -2.15 0.000

MMU05133.PERTUSSIS.KEGG -2.14 0.000

MMU04670.LEUKOCYTE.TRANSENDOTHELIAL.MIGRATION.KEGG -2.14 0.000

MMU05320.AUTOIMMUNE.THYROID.DISEASE.KEGG -2.14 0.000

MMU04612.ANTIGEN.PROCESSING.AND.PRESENTATION.KEGG -2.10 0.000

MMU05322.SYSTEMIC.LUPUS.ERYTHEMATOSUS.KEGG -2.07 0.000

MMU05310.ASTHMA.KEGG -2.02 0.000

MMU04650.NATURAL.KILLER.CELL.MEDIATED.CYTOTOXICITY.KEGG -1.99 0.000

MMU05321.INFLAMMATORY.BOWEL.DISEASE..IBD..KEGG -1.95 0.001

MMU04662.B.CELL.RECEPTOR.SIGNALING.PATHWAY.KEGG -1.94 0.001

MMU04064.NF.KAPPA.B.SIGNALING.PATHWAY.KEGG - 1.92 0.001

MMU04611.PLATELET.ACTIVATION.KEGG -1.91 0.001

MMU03030.DNA.REPLICATION.KEGG -1.89 0.002

MMU03010.RIBOSOME.KEGG -1.88 0.002

MMU04210.APOPTOSIS.KEGG -1.87 0.002

MMU00100.STEROID.BIOSYNTHESIS.KEGG -1.87 0.002

MMU05144.MALARIA.KEGG -1.85 0.003

MMU04621.NOD.LIKE.RECEPTOR.SIGNALING.PATHWAY.KEGG -1.82 0.004

MMU05169.EPSTEIN.BARR.VIRUS.INFECTION.KEGG -1.82 0.004

MMU05142.CHAGAS.DISEASE..AMERICAN.TRYPANOSOMIASIS..KEGG -1.81 0.004

MMU05164.INFLUENZA.A.KEGG -1.79 0.006

MMU00511.OTHER.GLYCAN.DEGRADATION.KEGG -1.77 0.006

MMU05202.TRANSCRIPTIONAL.MISREGULATION.IN.CANCER.KEGG -1.75 0.008

MMU05418.FLUID.SHEAR.STRESS.AND.ATHEROSCLEROSIS.KEGG -1.73 0.010

MMU00480.GLUTATHIONE.METABOLISM.KEGG -1.73 0.011

MMU00603.GLYCOSPHINGOLIPID.BIOSYNTHESIS.GLOBO.AND.ISOGLOBO.SERIES.KEGG -1.72 0.011

MMU04115.P53.SIGNALING.PATHWAY.KEGG -1.72 0.011

MMU04110.CELL.CYCLE.KEGG -1.71 0.012

MMU05163.HUMAN.CYTOMEGALOVIRUS.INFECTION.KEGG -1.71 0.012

MMU04625.C.TYPE.LECTIN.RECEPTOR.SIGNALING.PATHWAY.KEGG -1.70 0.013

MMU05170.HUMAN.IMMUNODEFICIENCY.VIRUS.1.INFECTION.KEGG -1.69 0.014

MMU05143.AFRICAN.TRYPANOSOMIASIS.KEGG -1.68 0.015

MMU05167.KAPOSI.SARCOMA.ASSOCIATED.HERPESVIRUS.INFECTION.KEGG -1.67 0.017

MMU05221.ACUTE.MYELOID.LEUKEMIA.KEGG -1.67 0.017

MMU04620.TOLL.LIKE.RECEPTOR.SIGNALING.PATHWAY.KEGG -1.67 0.017

MMU04664.FC.EPSILON.RI.SIGNALING.PATHWAY.KEGG -1.67 0.017

MMU04971.GASTRIC.ACID.SECRETION.KEGG -1.66 0.018

MMU00600.SPHINGOLIPID.METABOLISM.KEGG -1.65 0.019

MMU04810.REGULATION.OF.ACTIN.CYTOSKELETON.KEGG -1.65 0.019

MMU04015.RAP1.SIGNALING.PATHWAY.KEGG -1.65 0.019

MMU05132.SALMONELLA.INFECTION.KEGG -1.64 0.020

MMU03440.HOMOLOGOUS.RECOMBINATION.KEGG -1.64 0.020

MMU05100.BACTERIAL.INVASION.OF.EPITHELIAL.CELLS.KEGG -1.64 0.020

MMU00520.AMINO.SUGAR.AND.NUCLEOTIDE.SUGAR.METABOLISM.KEGG -1.63 0.023

MMU04750.INFLAMMATORY.MEDIATOR.REGULATION.OF.TRP.CHANNELS.KEGG -1.62 0.023

MMU04924.RENIN.SECRETION.KEGG -1.61 0.025

MMU04610.COMPLEMENT.AND.COAGULATION.CASCADES.KEGG -1.59 0.031

MMU03430.MISMATCH.REPAIR.KEGG -1.56 0.040

MMU04740.OLFACTORY.TRANSDUCTION.KEGG -1.55 0.043

MMU04979.CHOLESTEROL.METABOLISM.KEGG -1.55 0.044

MMU00240.PYRIMIDINE.METABOLISM.KEGG -1.55 0.045

MMU04072.PHOSPHOLIPASE.D.SIGNALING.PATHWAY.KEGG -1.54 0.048

MMU00980.METABOLISM.OF.XENOBIOTICS.BY.CYTOCHROME.P450.KEGG -1.53 0.049

MMU05340.PRIMARY.IMMUNODEFICIENCY.KEGG -1.53 0.049

MMU04658.TH1.AND.TH2.CELL.DIFFERENTIATION.KEGG -1.53 0.050

MMU05145.TOXOPLASMOSIS.KEGG -1.53 0.050

MMU04970.SALIVARY.SECRETION.KEGG -1.52 0.050

MMU05166.HUMAN.T.CELL.LEUKEMIA.VIRUS.1.INFECTION.KEGG -1.51 0.056

MMU04933.AGE.RAGE.SIGNALING.PATHWAY.IN.DIABETIC.COMPLICATIONS.KEGG -1.51 0.056

MMU04540.GAP.JUNCTION.KEGG -1.50 0.059

MMU00340.HISTIDINE.METABOLISM.KEGG -1.50 0.059

MMU04071.SPHINGOLIPID.SIGNALING.PATHWAY.KEGG -1.50 0.058

MMU00533.GLYCOSAMINOGLYCAN.BIOSYNTHESIS...KERATAN.SULFATE.KEGG -1.49 0.061

MMU04510.FOCAL.ADHESION.KEGG -1.48 0.068

MMU00590.ARACHIDONIC.ACID.METABOLISM.KEGG -1.48 0.068

MMU05014.AMYOTROPHIC.LATERAL.SCLEROSIS..ALS..KEGG -1.47 0.070

MMU04218.CELLULAR.SENESCENCE.KEGG -1.47 0.071

MMU04914.PROGESTERONE.MEDIATED.OOCYTE.MATURATION.KEGG -1.45 0.081

MMU05200.PATHWAYS.IN.CANCER.KEGG -1.45 0.081

MMU00513.VARIOUS.TYPES.OF.N.GLYCAN.BIOSYNTHESIS.KEGG -1.45 0.083

MMU04973.CARBOHYDRATE.DIGESTION.AND.ABSORPTION.KEGG -1.44 0.085

MMU05146.AMOEBIASIS.KEGG -1.44 0.087

MMU05134.LEGIONELLOSIS.KEGG -1.44 0.086

MMU05135.YERSINIA.INFECTION.KEGG -1.43 0.088

MMU00053.ASCORBATE.AND.ALDARATE.METABOLISM.KEGG -1.43 0.089

MMU05032.MORPHINE.ADDICTION.KEGG -1.43 0.091

MMU00514.OTHER.TYPES.OF.O.GLYCAN.BIOSYNTHESIS.KEGG -1.42 0.093

MMU04520.ADHERENS.JUNCTION.KEGG -1.42 0.093
