# Supplementary material for: RNA sequencing reveals niche gene expression effects of beta-hydroxybutyrate in primary myotubes
Source: Life Sci Alliance. 2021 Aug 18;4(10):e202101037. doi: 10.26508/lsa.202101037 (PMC8380668; doi:10.26508/lsa.202101037)
Supplement: Supplementary file 4 [file LSA-2021-01037_TableS4.docx]

**Supplemental table 4: Primers for qPCR**

| **Gene** | **Forward primer** | **Reverse primer** |
| --- | --- | --- |
| mBdh1 | ACAAGACACACGCTGTTGTTT | CTCTTCAAGCTGTCCAGTTCC |
| mMct1 | TGTTAGTCGGAGCCTTCATTTC | CACTGGTCGTTGCACTGAATA |
| mMct2 | GCTGGGTCGTAGTCTGTGC | ATCCAAGCGATCTGACTGGAG |
| mMct6/7 | ACATCTCCATAGGGGTAATCTCG | TGACTACCGAACGCCTTTTGT |
| mOxct1 | CATAAGGGGTGTGTCTGCTACT | GCAAGGTTGCACCATTAGGAAT |
| mDgat2 | TTCCTGGCATAAGGCCCTATT | AGTCTATGGTGTCTCGGTTGAC |
| mApoc2 | AGCTGCCTGATATGGAAGGA | GCTGTCTGGGACCTGAATGT |
| mGadph | CATGTTCCAGTATGACTCCACTC | GGCCTCACCCCATTTGATGT |
| mInppl1 | CAGCCTGGTATCACCGTGAC | GCCACGCTCTCGCTATCTC |
| mH1f0 | CACGGACCACCCCAAGTATTC | ACCCACCTTGTAGTGGCTCT |
| mPhlda3 | CCGTGGAGTGCGTAGAGAG | TCTGGATGGCCTGTTGATTCT |
| mClec4a1 | GACTCGTCTTCATGTACCGTCT | AGCAACAGAGAATAAGATTGCCA |
| mAdipoq | GCAGAGATGGCACTCCTGGA | CCCTTCAGCTCCTGTCATTCC |
| mSlc2a4 | GGAAGGAAAAGGGCTATGCTG | TGAGGAACCGTCCAAGAATGA |
| mFabp4 | AAGGTGAAGAGCATCATAACCCT | TCACGCCTTTCATAACACATTCC |
| mMyoD | GCCGCCTGAGCAAAGTGAATG | CAGCGGTCCAGGTGCGTAGAAG |
| mMyoG | AGAGGAAGTCTGTGTCGGTG | GTAGGCGCTCAATGTACTGG |
| mMyf5 | AAGGCTCCTGTATCCCCTCAC | TGACCTTCTTCAGGCGTCTAC |
| mMyh1 | GCGAATCGAGGCTCAGAACAA | GTAGTTCCGCCTTCGGTCTTG |
| mMyh2 | CGAAGAGTAAGGCTGTCCCG | CACAGGCGCATGACCAAAG |
| mMyh3 | AAAAGGCCATCACTGACGC | CAGCTCTCTGATCCGTGTCTC |
| mMyh4 | TTGAAAAGACGAAGCAGCGAC | AGAGAGCGGGACTCCTTCTG |
| mMyh7 | ACTGTCAACACTAAGAGGGTCA | TTGGATGATTTGATCTTCCAGGG |
| mMyh8 | AACAGAAACGCAATGCTGAGG | TCGCCTGTAATTTGTCCACCA |
| mAdipoq | TCCGTAAACATTTCCGGCCC | CAACTCGCAGGCTCAACTCC |
| mSlc2a4 | GGAAGGAAAAGGGCTATGCTG | TGAGGAACCGTCCAAGAATGA |
| mFabp4 | GGTGGAATGTGTTATGAA | AAATCCGACTGACTATTG |
| hCD11b | ACTTGCAGTGAGAACACGTATG | TCATCCGCCGAAAGTCATGTG |
| hCD14 | ACGCCAGAACCTTGTGAGC | GCATGGATCTCCACCTCTACTG |
| hTLR4a | TACAAAATCCCCGACAACCTCC | GCTGCCTAAATGCCTCAGGG |
| hTLR4b | ATGCTGCCGTTTTATCACGGA | CTAAACTCTGGATGGGGTTTCC |
| hLPL | CATTCCCGGAGTAGCAGAGT | GGCCACAAGTTTTGGCACC |
| hCD68 | GCTACATGGCGGTGGAGTACAA | ATGATGAGAGGCAGCAAGATGG |
| hTNFa | ATGAGCACTGAAAGCATGATCC | GAGGGCTGATTAGAGAGAGGTC |
| hCD36 | GTGCTGTCCTGGCTGTGTTT | TCACTTCCTGTGGATTTTGC |
| hIL1b | CACGATGCACCTGTACGATCA | GTTGCTCCATATCCTGTCCCT |
| hCD163 | TTCGTCGCATTATTCTTCTTGACTAA | TGGTGGACTAAGTTCTCTCCTCTTG |
| hSEPP1 | TCTGCCCGAAGTCCCTGTC | CTGATGCTGCCATTGTCGAC |
| hSTAB1 | TGCCCGCAAGAACCTCTC | GATCCCGTCACCCACGAAC |
| hCMKLR1 | CAGTTACGGTGATGAATACCCTG | GACGATGCTGTAGACCACCAC |
